# Type 2 diabetes is linked to higher physiologic markers of effort during exercise

**DOI:** 10.3389/fcdhc.2024.1346716

**Published:** 2024-04-29

**Authors:** Amy G. Huebschmann, Rebecca L. Scalzo, Xinyi Yang, Sarah J. Schmiege, Jane E. B. Reusch, Andrea L. Dunn, Kristina Chapman, Judith G. Regensteiner

**Affiliations:** ^1^ Division of General Internal Medicine, University of Colorado, Aurora, CO, United States; ^2^ Ludeman Family Center for Women’s Health Research, University of Colorado, Aurora, CO, United States; ^3^ Adult and Child Center for Outcomes Research and Delivery Science, University of Colorado, Aurora, CO, United States; ^4^ Division of Endocrinology, University of Colorado, Aurora, CO, United States; ^5^ Eastern Colorado Veterans Administration Medical Center, Aurora, CO, United States; ^6^ Department of Biostatistics and Informatics, Colorado School of Public Health, University of Colorado, Aurora, CO, United States; ^7^ Senior Scientist Emeritus, Klein-Buendel, Inc., Golden, CO, United States

**Keywords:** type 2 diabetes mellitus, exercise, physical exertion (MeSH term), cardiorespiratory fitness (MeSH term), lactate, heart rate

## Abstract

**Background:**

People with type 2 diabetes (T2D) have lower rates of physical activity (PA) than the general population. This is significant because insufficient PA is linked to cardiovascular morbidity and mortality, particularly in individuals with T2D. Previously, we identified a novel barrier to physical activity: greater perceived effort during exercise in women. Specifically, women with T2D experienced exercise at low-intensity as greater effort than women without T2D at the same low-intensity – based on self-report and objective lactate measurements. A gap in the literature is whether T2D confers greater exercise effort in both sexes and across a range of work rates.

**Objectives:**

Our overarching objective was to address these gaps regarding the influence of T2D and relative work intensity on exercise effort. We hypothesized that T2D status would confer greater effort during exercise across a range of work rates below the aerobic threshold.

**Methods:**

This cross-sectional study enrolled males and post-menopausal females aged 50-75 years. Measures of exercise effort included: 1) heart rate, 2) lactate and 3) self-report of Rating of Perceived Exertion (RPE); each assessment was during the final minute of a 5-minute bout of treadmill exercise. Treadmill exercise was performed at 3 work rates: 1.5 mph, 2.0 mph, and 2.5 mph, respectively. To determine factors influencing effort, separate linear mixed effect models assessed the influence of T2D on each outcome of exercise effort, controlling for work rate intensity relative to peak oxygen consumption (%VO_2_peak). Models were adjusted for any significant demographic associations between effort and age (years), sex (male/female), baseline physical activity, or average blood glucose levels.

**Results:**

We enrolled n=19 people with T2D (47.4% female) and n=18 people (55.6% female) with no T2D. In the models adjusted for %VO_2_peak, T2D status was significantly associated with higher heart rate (p = 0.02) and lactate (p = 0.01), without a significant association with RPE (p = 0.58).

**Discussions:**

Across a range of low-to-moderate intensity work rates in older, sedentary males and females, a diagnosis of T2D conferred higher objective markers of effort but did not affect RPE. Greater objective effort cannot be fully attributed to impaired fitness, as it persisted despite adjustment for %VO_2_peak. In order to promote regular exercise and reduce cardiovascular risk for people with T2D, 1) further efforts to understand the mechanistic targets that influence physiologic exercise effort should be sought, and 2) comparison of the effort and tolerability of alternative exercise training prescriptions is warranted.

## 1 Introduction

The prevalence of type 2 diabetes (T2D) continues to rise; as of 2022, more than 1 in 10 adults in the United States had T2D (1). Exercise is a cornerstone of treatment for people with T2D, due to its multiple benefits, including improved glycemic control, fitness, and cardiovascular health (2, 3). However, people with type 2 diabetes (T2D) have lower rates of physical activity (PA) than the general population (2, 4). In addition, among adults with T2D, females are less active than males (4). Low activity among people with T2D is a serious health problem because insufficient PA is linked to higher rates of morbidity and premature mortality due to cardiovascular disease, cancer, and disability (2, 3).

Our prior research has identified a novel barrier to physical activity: greater perceived effort during exercise in women (5, 6). In particular, we found that women with T2D experience exercise at low-intensity work rates as greater effort than similarly sedentary and overweight women without T2D — based on self-report (5) and objective lactate (6) measurements. This finding is consistent with earlier work by our laboratory and others that found T2D is linked to impairment of cardiorespiratory fitness by ~10-15%, as measured by the gold standard assessment of peak oxygen consumption (VO_2_peak) (7–12). In turn, impairment in fitness confers a greater relative work intensity for a given work rate, thus increasing both objective markers of exercise effort and the rate of perceived exertion (10–12). Uncomfortable levels of effort during exercise play a significant role in adherence to physical activity prescriptions (13–15). Given that effort during exercise influences physical activity and is modifiable, it has great potential relevance as a barrier to physical activity that may be treated (16–19). In addition, disparities in effort during exercise may explain some prior barriers to physical activity specifically voiced by people with T2D, such as “difficulty keeping up with others who don’t have diabetes” (20).

There are several potential influences of effort during exercise for people with T2D. In the literature, exercise effort during equivalent absolute work rates has been shown to be influenced by cardiorespiratory fitness (15, 21). In turn, cardiorespiratory fitness in T2D — as measured by VO_2_peak — is influenced by several factors, including: chronological age and impairments in vascular elasticity and endothelial function, cardiac function, and mitochondrial function (22, 23). Also, it is possible that biological sex may moderate the influence of T2D on exercise effort, given that our prior work found that the presence of T2D conferred a greater impairment of VO_2_peak on females than males (9). A gap in the literature is whether T2D confers greater exercise effort in both sexes and across a range of work rates.

Our overarching objective in this pilot study was to address the gap in the literature regarding the influence of relative work intensity on exercise effort in a mixed sample of women and men with T2D, as our prior studies demonstrating T2D conferred increased effort were limited to women. We also sought to explore potential demographic factors, including the role of sex as a biological variable given that the presence of T2D confers a greater impairment of VO_2_peak for females than males (9). Our premise was that if there are no longer significant differences in effort between people with and without T2D after accounting for the lower fitness levels in people with T2D, then standard exercise training tailored to a relative percentage of one’s peak heart rate or VO_2_peak should be appropriate. In contrast, if exercise effort is still greater in people with T2D at equivalent relative work rates, this would suggest that other mechanistic factors driving effort during exercise need to be identified and addressed to improve exercise adherence. Our first aim (Aim 1a) was to determine the association between T2D and exercise effort across varying absolute work rates below the ventilatory threshold. Due to the known impairment of VO_2_peak in individuals with T2D (23), our hypothesis for this aim was: across equivalent absolute work rates below the ventilatory threshold, there is greater exercise effort during exercise in participants with T2D compared to those without diabetes. Our second aim (Aim 1b) was to determine the association between T2D and exercise effort at equivalent relative work rates that were adjusted for the relative exercise intensity as compared to that individual’s VO_2_peak. As there are other affective factors as well as physiologic factors that have been linked to effort during exercise in patients with obesity/T2D in addition to VO_2_peak, we hypothesized that across equivalent relative work rates below the ventilatory threshold, there is greater exercise effort during exercise in participants with T2D compared to those without diabetes (15, 18, 19, 21, 23). Finally, we explored whether sex as a biological variable, age, baseline physical activity, or average blood sugar levels (i.e., hemoglobin A1c) influenced the outcome of exercise effort in our study sample.

## 2 Materials and methods

### 2.1 Study design

This cross-sectional study enrolled males and post-menopausal females aged 50-75 years with and without T2D between 2012-2018. Over a period of 2 months, consented participants completed research eligibility screening visits, and those who met eligibility criteria performed a series of exercise testing visits in our exercise laboratory. The study was approved as human subjects research by the Colorado Multiple Institutional Review Board (COMIRB).

### 2.2 Recruitment

Participants were identified from community and clinical care settings, using advertisements in local newspapers, community centers and in clinics within the University of Colorado Health system. An initial phone screening questionnaire was used to identify interested participants who appeared eligible based on the inclusion criteria. Interested participants completed an in-person informed consent and completed a medical history and physical with further laboratory and other screening tests at an initial research eligibility screening visit. Eligible participants after the initial screening visit completed a second research screening visit, including a maximal exercise treadmill test to rule out participants with ischemic electrocardiogram changes during exercise. The following inclusion and exclusion criteria were assessed over both research screening visits — participants that met these criteria were enrolled in the study.

• Inclusion criteria:

o Age = 50-75 years (Justification: this range was selected to be inclusive of post-menopausal middle-aged to older women and age-similar men; in terms of the upper limit of age, we selected age 75 years as an age cutoff above which our participants were more likely to experience functional impairments with exercise due to arthritis).
o Body Mass Index (BMI) = 25-35 kg/m^2^ (Justification: this range was selected in order to include both overweight and obese participants, but to avoid the potential influence of more severe obesity to alter the biomechanics of gait and thus confound effort).
o In women, post-menopausal based on both follicle stimulating hormone > 15 mg/dl and no menses for 1 year.
o Sedentary, by leisure physical activity ≤ 60 minutes weekly, assessed by the Low Physical Activity Recall (LOPAR) survey (24).
o For healthy controls ONLY: normoglycemia – fasting glucose < 100 mg/dl and hemoglobin A1c < 5.7% (25).
o For patients with T2D ONLY: diagnosis of T2D within the last 15 years, no microvascular complications of diabetes, reasonable glycemic control with hemoglobin A1c < 8.0%, and the only allowable medications to take for diabetes were metformin or sulfonylureas.

• Exclusion criteria used in our prior studies of exercise in patients with T2D (7, 26) – assessed by medical history and physical by a research-trained physician:

o Medications that lower heart rate (beta blockers or calcium channel blockers), as heart rate is one of the assessments of exercise effort.
o Estrogen hormone replacement therapy.
o Uncontrolled hypertension > 140/90 mm Hg at rest or > 250/115 mm Hg during maximal exercise testing.
o Atherosclerotic disease (e.g., history of coronary artery disease, cerebrovascular disease, peripheral artery disease (PAD)) – assessed by maximal exercise treadmill test and screening ankle brachial index.
o Chronic obstructive pulmonary disease (assessed by pulmonary function test as the ratio of forced expiratory volume in 1 second over forced vital capacity < 0.7).
o Exertional musculoskeletal symptoms.
o Smoking within last 1 year.
o Chronic kidney or liver disease (assessed by laboratory testing – abnormal creatinine clearance or liver function tests).
o Autonomic dysfunction.
o Clinical evidence of cognitive dysfunction (assessed by Folstein Mini-Mental Status exam score < 25).

### 2.3 Study setting

All study visits took place at the University of Colorado’s NIH-funded Clinical Translational Research Center. All study procedures, including submaximal and maximal testing, were conducted by our experienced research assistants. A physician supervised the initial maximal exercise test.

### 2.4 Description of study exercise effort outcome variables

#### 2.4.1 Primary outcome variable: Borg rating of perceived exertion

RPE is the gold standard measure of perceived effort during exercise in both healthy populations (2, 21, 27) and populations with T2D (28). The range of scores on this scale is from 6-20. Higher scores on the RPE scale represent greater perceived exercise effort.

• Lactate level was measured using the lactate dehydrogenase method on serum drawn in perchloric acid tubes (7).
• Heart rate (beats per minute) was measured by the heart rate assessed by the metabolic cart (Medgraphics Ultima CPX metabolic cart, Minneapolis, MN), generated from assessment by the 12-lead ECG leads.

#### 2.4.2 Description of potential covariates

• Cardiorespiratory Fitness (VO_2_peak) was measured during maximal exercise treadmill tests with breath-by-breath VO_2_ and VCO_2_ (Medgraphics Ultima CPX metabolic cart, Minneapolis, MN), blood pressure (auscultation), and cardiac status (12-lead electrocardiogram). To determine VO_2_peak, subjects exercised to exhaustion using a standard protocol as previously described (7, 8, 26). During incremental exercise testing, the highest VO_2_ and heart rate averaged over 20 seconds were defined as the peak values. VO_2_peak was confirmed by a respiratory exchange ratio (RER) greater than 1.10 and VO_2_ plateau during the final minute of exercise. Based on the VO_2_peak test, we also identified the Ventilatory Threshold (VT) for each participant using the V-slope method (10).. Briefly, VT is defined as the point of non-linear increase in expired CO_2_ (VCO_2_), and assessing this for each participant was relevant in order to exclude data from work rates that were not at steady-state (i.e., above VT).
• Age (years) was assessed during the medical history and physical visit.
• Sex (male/female) was assessed during the medical history and physical visit.

### 2.5 Study visits

**Figure 1:**
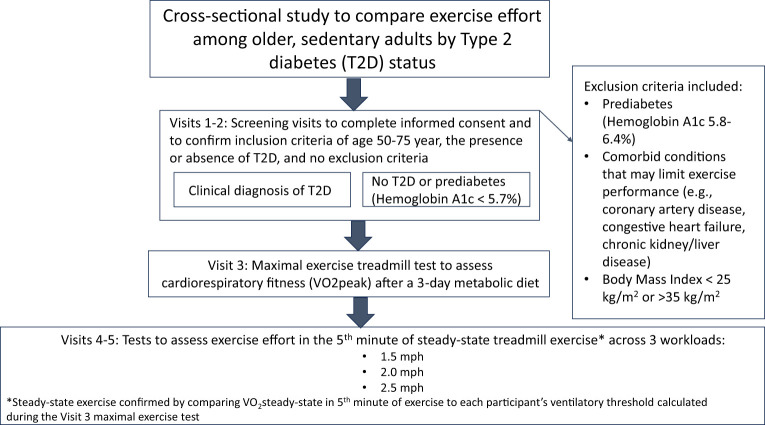
Visual summary of the main activities during each study visit.

A brief narrative of each study visit is provided, as well as a visual summary depiction of the major activities from each visit (**Figure 1**).

• (Visits 1 and 2 – eligibility screening visits): Informed consent and medical screening visit which includes obtaining informed consent and completing the LOPAR survey and screening laboratory tests needed to assess the inclusion/exclusion criteria. All participants performed a maximal exercise stress test to rule out ischemia, and completed pulmonary function tests and autonomic nervous system tests to confirm inclusion/exclusion criteria. The study physician performed a medical history and physical examination to confirm inclusion/exclusion criteria. Participants also completed a dietary survey and performed a bone densitometry test (DEXA) to obtain the body composition measures for VO_2_peak, and received an accelerometer to wear for 1 week.
• Study diets for Visits 3-5: For optimal performance, meals with 45% carbohydrate, 35% fat, and 20% protein content matched participants’ caloric needs for the 3 days immediately prior to exercise testing. Caloric needs were determined from participants’ body composition by DEXA. Participants also fasted for 4 hours prior to performing exercise on visits 3-5.
• Visit 3: Participants performed a maximal treadmill exercise test to assess VO_2_peak.
• Visits 4-5: Exercise testing and blood draws were identical during Visits 4 and 5, as it is an excessive participant burden to complete these tests in one day. On both study days, participants performed one 5-minute bout of treadmill exercise at each of three constant work rates. The work rates were performed in order of ascending difficulty, beginning at low intensity (1.5 mph and 2 mph at 0% grade), then progressing to moderate intensity (2.5 mph at 0% grade). To compare to our prior studies of effort during cycle ergometer exercise, 2 mph at 0% grade of treadmill exercise is comparable to 30W of cycle ergometer exercise. Resting blood pressure and heart rate were assessed and serum lactate levels were drawn from an IV at rest before the first exercise bout. At the beginning of the 5^th^ minute of each exercise bout, participants rated RPE, serum lactate levels were drawn from the IV, and heart rate was assessed.

### 2.6 Analysis

All statistical analyses were carried out using R Version 3.6.2 and SAS version 9.3. Our analyses sought to test these two hypotheses:

Hypothesis 1a: Across equivalent absolute work rates below the Ventilatory Threshold (VT), there is greater effort during exercise in participants with T2D compared to those without diabetes.
Hypothesis 1b: Across equivalent relative work rates below the VT, there is greater effort during exercise in participants with T2D compared to those without diabetes.

The analytic approach first sought to confirm all data from both visit 4 and visit 5 were obtained at steady-state work rates below the VT, in accordance with our specific aims. In brief, we compared the oxygen consumption (VO_2_) at the individual’s work rates during the submaximal tests during Visits 4 and 5 to the VO2 at the individual’s VT. The VO_2_ that corresponded with an individual’s VT was used to determine if each work rate was above or below VT. The rationale for this was that effort at work rates above VT should not be included as it would not be at steady-state. This was a 2-step process. We first identified 2 categories for each bout of submaximal exercise: 1) “definitely below VT” – based on all four 15-second VO_2_ averages in the final minute of exercise being below VT; 2) “possibly above VT” – based on at least one mean VO_2_ in the final minute being above the VT. For the “possibly above VT” measures, two independent study team members (KC and AGH) conducted a qualitative assessment to clarify if the breath-by-breath VO_2_ had plateaued in the 5^th^ minute of the bout or not. If there was breath-by-breath VO_2_ plateau, then we recategorized this work rate as “definitely below VT”, but if there was no plateau, we excluded the effort data on that work rate from the analyses to test hypotheses 1a and 1b.

Prior to hypothesis testing, descriptive statistics were calculated on study variables using means and standard deviations (SDs) for continuously measured variables and proportions for categorically measured variables. Data from visits 4 and 5 were averaged for all statistical modeling after ensuring consistency in responses of all predictors/outcomes between study visits, including confirmation that VO^2^ data used in the model were obtained at steady-state workloads below VT as described above. Consistency in responses between visits was confirmed through mixed effects regression models that included visit as a factor (main effect and interaction with T2D status) predicting each outcome and observing no significant differences by visit (p values ranged from 0.36 to 0.96). Mixed effect regression models were estimated to determine the association of T2D with exercise effort across equivalent absolute work rates (Hypothesis 1a) and relative work rates (Hypothesis 1b), using an alpha = 0.05.

The same general modeling process was followed for both hypotheses, whereby the primary association of interest was of T2D status with each of the three outcomes of exercise effort (i.e., heart rate, lactate, and RPE). Separate linear mixed models were developed for each of the three outcomes (six models total). Models were estimated that had the same general form of time/bout, T2D status, and the time by T2D status interaction as the fixed effects of interest. Mixed effects regression models were used so that a subject-level random effect could be included to account for the repeated measures data structure of bout nested within patients (29). A time by T2D status interaction signifies that the impact of absolute work rate (Hypothesis 1a) or relative work rate (Hypothesis 1b) on exercise efforts over the three exercise bouts differed for patients with T2D vs. without T2D. In all instances, the interaction term was non-significant and lower order terms were interpreted accordingly as the average difference between those with T2D and without T2D, and the average impact of absolute work rate (Hypothesis 1a) or relative work rate (%VO_2_peak; Hypothesis 1b) over time on exercise effort.

Age, baseline physical activity, sex, and average glucose levels (i.e., Hemoglobin A1c) were tested as potential covariates in all models, and were only retained in the model if they significantly impacted exercise effort outcomes (p < 0.05); most covariates were therefore trimmed from the final models for parsimony, however, conclusions of the primary effects of interest were consistent across unadjusted and adjusted models. A subject-level random effect was modeled to account for the repeated measures data structure of bouts nested within patients. Only work rates below the VT were used in modeling, and we required at least 50% of subjects within each group to have steady state VO_2_ data below VT available to include a given bout in analyses; this resulted in sufficient data coverage to use the three bouts of exercise at 1.5 mph, 2.0 mph and 2.5 mph in these analyses.

## 3 Results

We enrolled n=19 people with T2D (47.4% female) and n=18 people (55.6% female) with no T2D who met our study eligibility criteria (**Table 1**). There were no differences in the distribution of age, sex or race/ethnicity by T2D status. The group with T2D was more likely to have a diagnosis of hypertension (p = 0.001) and had higher average glucose levels based on Hemoglobin A1c (p < 0.001) than those without T2D. As expected from our prior work, cardiorespiratory fitness was significantly worse (p < 0.001) in those with T2D (21.8 ± 3.2 ml/kg/min) vs. overweight, sedentary controls with no T2D (24.8 ± 5.8 ml/kg/min). Both study groups (i.e., with T2D and without T2D) achieved criteria for a valid VO_2_peak.

**Table 1:** Demographic and physiologic measures.

	Overweight sedentary controls without T2D n=19	Type 2 diabetes (T2D) n = 18	P-value
**Age (years)**	60.6 (6.2)	61.9 (5.4)	P = 0.49
**% Female**	47.4	55.6	P = 0.87
**Race/Ethnicity**	84.2% White 5.3% Black 0% Asian 5.3% America Indian or Alaska Native 10.5% Hispanic	55.6% White 16.7% Black 11.1% Asian 0% America Indian or Alaska Native 11.1% Hispanic	P = 0.12 for white vs. non-white; P = 0.803 for Hispanic vs. not Hispanic
**Body Mass Index (kg/m^2^)**	30.05 (2.8)	31.58 (3.5)	P = 0.16
**Hypertension diagnosis (%yes)**	5.3	61.1	P = 0.001
**Hemoglobin A1c**	5.3 (0.3)	6.6 (0.7)	P < 0.001
**Use of metformin to treat T2D**	0%	61%	P < 0.001
**Self-reported physical activity (Metabolic Equivalent (MET)-hours per week)**	242 (25)	232 (38)	P = 0.375
**Objective physical activity (counts)**	871 (86)	916 (55)	P = 0.07
**Peak oxygen consumption (VO_2_peak) (ml/kg/min)**	24.8 (5.8)	21.8 (3.2)	P <0.001
**Respiratory Exchange Ratio at VO_2_peak**	1.13 (0.12)	1.13 (0.09)	P = 0.86

Data presented as % (categorical data) or as mean (SD) for continuous data.

Figure titles are provided first, followed by a thumbnail graphic, and followed by the caption. There are separate graphics files uploaded and these thumbnails are only included for the ease of reviewers.

### 3.1 Assessment of the influence of T2D on effort during exercise

**Figure 2:**
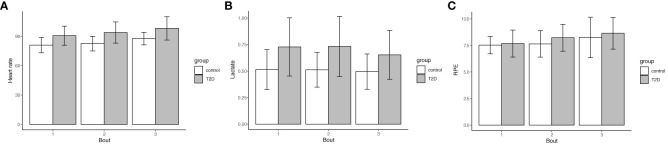
Type 2 Diabetes status confers higher heart rate and lactate levels during exercise, but no significant difference in RPE. The assessment of effort as heart rate **(A)**, lactate levels **(B)** and RPE **(C)** during exercise were averaged across 3 treadmill bouts of 1.5 mph, 2.0 mph, and 2.5 mph. Data are shown as mean (bar graph) and standard deviations (error bars) are stratified by bout and Type 2 Diabetes status (n=18 with T2D, 55.6% female; no T2D (n=19, 47.4% female). **(A)** (heart rate) Bout 1: 90.6 (9.8) vs. 81.0 (7.8), Bout 2: 93.9 (10.8) vs. 82.6 (7.5), Bout 3: 98.1 (11.9) vs. 87.7 (6.4), p=0.002 averaged across bouts; **(B)** (lactate) Bout 1: 0.73 (0.27) vs. 0.51 (0.19), Bout 2: 0.73 (0.28) vs. 0.51 (0.16), Bout 3: 0.65 (0.23) vs. 0.50 (0.17), p = 0.009 averaged across bouts; **(C)** (RPE) Bout 1: 7.7 (1.3) vs. 7.5 (0.82), Bout 2: 8.2 (1.3) vs. 7.6 (1.3), Bout 3: 8.6 (1.5) vs. 8.3 (1.9), p = 0.75 averaged across bouts. The reported p-values were appropriate to average across all 3 bouts for each of the models because there was no effect modification by bout of the association between T2D status and effort.

In the Hypothesis 1a models used to test the influence of T2D on effort at absolute work rates, people with T2D had a significantly higher heart rate (**Figure 2A**, p = 0.002 averaged across all 3 bouts), and a significantly higher lactate level (**Figure 2B**, p = 0.009 averaged across all 3 bouts). RPE was not significantly higher across the 3 bouts of exercise (**Figure 2C**, p = 0.75 averaged across all 3 bouts). Of note, the reported p-values were appropriate to average across all 3 bouts for each of the models because there was no effect modification by bout of the association between T2D status and effort.

**Figure 3:**
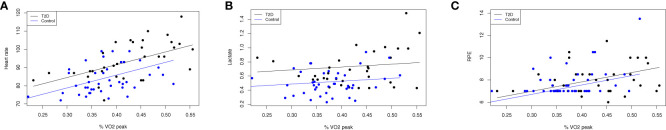
Differences in objective exercise effort persist after adjustment for relative work intensity. Data are displayed as scatter plots between effort (y-axis) of heart rate **(A)**, lactate levels **(B)** and RPE **(C)** during exercise and the relative work intensity (x-axis) of the percentage of peak oxygen consumption displayed as a fraction (i.e., 0.30 = 30% VO_2_peak) during each bout of 1.5 mph, 2.0 mph and 2.5 mph. Identical participants as shown in **Figure 2**: (T2D) status (n=18 with T2D, 55.6% female; no T2D (n=19, 47.4% female). In these adjusted models, the p-values were attenuated with adjustment for the relative work intensity; however, T2D status was still significantly associated with higher heart rate (p=0.02) and lactate (p=0.01), respectively, but there remained no significant association with RPE (p=0.58).

In the Hypothesis 1b models, we used the same data points as for Aim 1a. However, this analysis compared the association of T2D status and effort with adjustment for the relative work intensity of %VO_2_peak during each bout (**Figure 3**). In these adjusted models, the p-values were attenuated with adjustment for the relative work intensity; however, T2D status was still significantly associated with higher heart rate (p = 0.02) and lactate (p = 0.01), respectively, as shown in **Figure 3**. There remained no significant association with RPE (p = 0.58). In an exploratory analysis, we did not find any association between sex, baseline physical activity, or average glucose levels (HbA1c) and effort, so in accordance with our analytic plan these were trimmed from the final models. Higher age (years) was significantly associated with a higher heart rate during exercise (P = 0.02), but was not associated with either heart rate or RPE. Accordingly, the data presented here from model 1b.1 for heart rate were adjusted for age.

## 4 Discussion

Across a range of 3 low-to-moderate intensity work rates (1.5-2.5 mph) of treadmill exercise in older males and post-menopausal female individuals with and without T2D, the presence of T2D conferred higher objective markers of effort of heart rate and lactate, but did not influence RPE. These findings of greater objective effort during exercise cannot be fully attributed to impaired fitness levels, as the association between T2D and heart rate and lactate was still statistically significant after adjustment for %VO_2_peak – albeit partly attenuated. In sum, our findings for heart rate and lactate supported our hypotheses that there would be greater effort during exercise at both absolute and relative work rates in people with T2D as compared to no diabetes, but our findings for RPE ran counter to both of these hypotheses.

Our findings need to be placed in context of the prior work of our team and others – these data are the first (to our knowledge) to demonstrate objective exercise effort at pre-specified absolute and relative work rates in both males and females with T2D as compared to their peers without diabetes. We and others previously found that mid-life to older adults with T2D have greater markers of objective effort during cycle ergometer exercise and during a 6-minute walk test (5, 6, 30) – these data are the first (to our knowledge) to demonstrate objective exercise effort at pre-specified absolute and relative work rates in both males and females with T2D as compared to their peers without diabetes. Thus in contrast to our prior reports, these data show that both heart rate and lactate markers of effort are greater in both women and men, even at relatively low-intensity exercise. Data on RPE as a subjective marker of effort have been mixed, with one study in pre-menopausal women showing RPE is significantly higher in those with T2D vs. no T2D (5), and two studies showing no difference in RPE by T2D status (6, 30). We speculate that differences in objective effort may be observed before an individual becomes conscious of a higher level of perceived effort. Others have found that metformin influences heart rate during exercise; a recent meta-analysis found that the use of metformin for people with T2D leads to a modest but statistically significant increase in heart rate (31), and this is relevant as 61% of the patients with T2D in this study were taking metformin, whereas none of the control participants were taking this medicine. However, in one of our prior studies with greater objective effort during cycle ergometer exercise, none of the participants with T2D were taking metformin (5), so there appears to be factors outside of medication that influence disparate effort in those with T2D.

This study also explored the influence of sex as a biological variable, age, baseline physical activity and average glucose levels on effort. In the models that were adjusted for relative work intensity (%VO_2_peak), none of these covariates were retained for the models of lactate and RPE, and only age met criteria for retention in the model of heart rate. This was an exploratory analysis, so we need to be cautious with our conclusions, but it does not appear that there are different drivers of the higher levels of lactate and heart rate between the sedentary males and females studied, and the heart rate effort remained greater in those with T2D vs. no T2D even after adjustment for age. Future research should consider evaluating other demographic factors, comorbid conditions (e.g., hypertension, metformin/other medication use), and mechanistic moderators of the relationship between T2D and exercise effort, including insulin resistance, endothelial dysfunction and diastolic dysfunction (22, 32, 33).

The clinical implication of these data, and prior findings that effort during exercise is higher for people with T2D (5, 6, 30), is to lend further support to tailoring exercise goals to an individual’s exercise effort. This is in keeping with the published Physical Activity Guidelines for Americans in 2018 that supports that “all movement matters” to support health, from light to moderate to vigorous intensity exercise (3), and the recommendations by the American Diabetes Association to consider any movement as an initial “stepping stone” exercise prescription towards the recommended health goal of 150 minutes of weekly walking or other moderate intensity activity (2). In addition, a recent article suggested that exercise effort may explain >65% of the variance in activity in older adults, and suggested basing exercise prescriptions on heart rate or other objective physiologic effort for elders (33). Two small studies in individuals with T2D evaluated how the dose of exercise may influence effort and health (34, 35); specifically, Coquart et al. found that intermittent exercise generated lower RPE and HR as compared to a similar dose of continuous exercise; Viana et al. found that comparing high-intensity interval training with intensity based on either RPE or HR may have more salutary effects on blood pressure and glycemic control than a standard 30-minute continuous bout of exercise training (34, 35). Taken together, our findings in light of the published literature support further testing of alternate exercise prescriptions tailored to effort level on physiological outcomes and on exercise adherence, as compared to standard exercise training approaches.

This study has both strengths and limitations. A strength, as compared to our prior studies comparing effort in people with and without T2D during cycle ergometer exercise (5, 6), is that this study compared effort during treadmill exercise, and walking is the preferred activity for many adults with T2D (20, 33, 36). A limitation is that this pilot study did not have a sufficient sample size to model complex relationships, such as potential moderators of the association of T2D with exercise effort (e.g., sex as a biological variable, hypertension diagnosis, metformin use, insulin resistance, endothelial function, and diastolic dysfunction). There are also limitations in terms of the representativeness and generalizability of our findings, as our recruitment methods and study inclusion criteria led to the inclusion of a healthier cross-section of middle-aged to older patients with T2D than the general population with this disease — this limitation may have dampened the signal of increased exercise effort conferred by T2D.

In conclusion, this study adds to the literature showing that objective markers of effort during exercise are significantly higher in people with T2D as compared to those without diabetes (5, 6, 30). In order to promote regular exercise and reduce cardiovascular risk, further efforts to understand the mechanistic targets to reduce the modifiable barrier of physiologic exercise effort in T2DM should be sought. In addition, given that adherence to physical activity is lower when exercise effort is greater (33), further studies to compare both the effort/tolerability and health effects of novel exercise training strategies for patients with T2D are warranted — intermittent exercise prescriptions tailored to exercise effort (35) may be particularly promising. Our group is also considering the role of motivational interviewing-based behavioral interventions focused on identifying and doing enjoyable and meaningful physical activity (36), and high-resistance inspiratory muscle strength training (IMST) that has the potential to improve exercise tolerance, as well as cardiac and vascular function (37, 38).

## Data availability statement

The raw data supporting the conclusions of this article will be made available by the authors, without undue reservation.

## Ethics statement

The studies involving human subjects were approved by the Colorado Multiple Institutional Review Board (Approval #11-0909). The studies were conducted in accordance with the local legislation and institutional requirements. The participants provided their written informed consent to participate in this study.

## Author contributions

AH: Conceptualization, Funding acquisition, Methodology, Project administration, Writing – original draft, Writing – review & editing. RS: Conceptualization, Writing – review & editing. XY: Formal analysis, Visualization, Writing – review & editing. SS: Conceptualization, Formal analysis, Writing – review & editing. JEBR: Conceptualization, Writing – review & editing. AD: Conceptualization, Writing – review & editing. KC: Data curation, Project administration, Writing – review & editing. JGR: Conceptualization, Methodology, Supervision, Writing – review & editing.
